# Cellular Senescence in Health, Disease, and Lens Aging

**DOI:** 10.3390/ph18020244

**Published:** 2025-02-12

**Authors:** Ying Qin, Haoxin Liu, Hongli Wu

**Affiliations:** 1 Pharmaceutical Sciences, College of Pharmacy, University of North Texas Health Science Center, Fort Worth, TX 76107, USA; yingqin@my.unthsc.edu (Y.Q.); haoxinliu@my.unthsc.edu (H.L.); 2 North Texas Eye Research Institute, University of North Texas Health Science Center, Fort Worth, TX 76107, USA

**Keywords:** cellular senescence, lens epithelial cells, oxidative stress, cataractogenesis, senotherapeutics, senescence-associated secretory phenotype

## Abstract

**Background**: Cellular senescence is a state of irreversible cell cycle arrest that serves as a critical regulator of tissue homeostasis, aging, and disease. While transient senescence contributes to development, wound healing, and tumor suppression, chronic senescence drives inflammation, tissue dysfunction, and age-related pathologies, including cataracts. Lens epithelial cells (LECs), essential for maintaining lens transparency, are particularly vulnerable to oxidative stress-induced senescence, which accelerates lens aging and cataract formation. This review examines the dual role of senescence in LEC function and its implications for age-related cataractogenesis, alongside emerging senotherapeutic interventions. **Methods**: This review synthesizes findings on the molecular mechanisms of senescence, focusing on oxidative stress, mitochondrial dysfunction, and the senescence-associated secretory phenotype (SASP). It explores evidence linking LEC senescence to cataract formation, highlighting key studies on stress responses, DNA damage, and antioxidant defense. Recent advances in senotherapeutics, including senolytics and senomorphics, are analyzed for their potential to mitigate LEC senescence and delay cataract progression. **Conclusions**: LEC senescence is driven by oxidative damage, mitochondrial dysfunction, and impaired redox homeostasis. These factors activate senescence path-ways, including p53/p21 and p16/Rb, resulting in cell cycle arrest and SASP-mediated inflammation. The accumulation of senescent LECs reduces regenerative capacity, disrupts lens homeostasis, and contributes to cataractogenesis. Emerging senotherapeutics, such as dasatinib, quercetin, and metformin, show promise in reducing the senescent cell burden and modulating the SASP to preserve lens transparency.

## 1. Introduction

The life cycle of a cell is a dynamic and highly regulated process, driven by intricate molecular signals that guide its progression through various phases. From active proliferation to programmed death, cells continuously transition between states of growth, rest, repair, and death. Among these states, cellular senescence stands out as a critical regulatory mechanism. Defined as a state of irreversible cell cycle arrest, senescence is distinct from quiescence or temporary pauses in the cell cycle, ensuring that cells permanently cease division [1]. This process serves as a protective measure to prevent the spread of damaged DNA, thereby reducing the risk of cancer. Despite their inability to divide, senescent cells remain metabolically active and exhibit a notable feature known as the senescence-associated secretory phenotype (SASP). The SASP involves the secretion of inflammatory cytokines, growth factors, and proteases that can affect the surrounding tissue by promoting repair and regeneration [2]. However, when senescent cells accumulate or persist excessively, the SASP can shift from being beneficial to harmful, leading to chronic inflammation, tissue dysfunction, and age-related diseases [3]. Understanding cellular senescence and the SASP is crucial for recognizing its dual role in maintaining health and driving pathological conditions such as aging and degenerative diseases.

**Figure 1:**
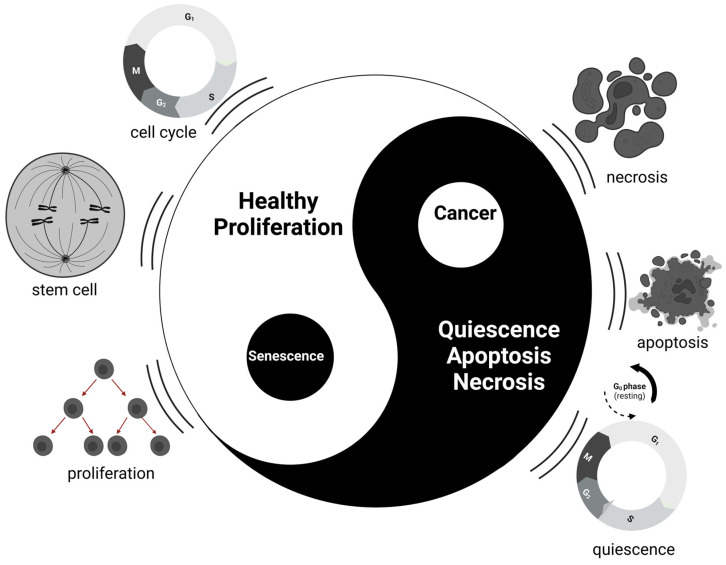
The Yin-Yang of Cellular Fate: Balancing Proliferation, Cell Death, and Senescence in Health and Disease. The Tai Chi (Yin-Yang) symbol represents the delicate equilibrium governing cellular fate by balancing proliferation, quiescence, and degeneration. The left (white, Yang) half symbolizes cellular growth, renewal, and division, driven by the active phases of the cell cycle (G1, S, G2, and M). Stem cells and proliferative cells inhabit this domain, reflecting their role in tissue regeneration and repair. In contrast, the right (black, Yin) half represents growth-suppressing forces, including quiescence (G0 phase), apoptosis, and necrosis—processes that ensure cellular rest, programmed cell death, and the clearance of damaged cells to preserve tissue integrity. The small black dot within the white half signifies senescent cells, which, while no longer dividing, remain metabolically active and shape their environment through secretory activity, contributing to inflammation, fibrosis, and tissue remodeling. Conversely, the white dot within the black half represents cancer cells, which escape from quiescent states to proliferate uncontrollably, disrupting cellular homeostasis. This figure encapsulates the dynamic interplay between proliferation and arrest, underscoring senescence as a key mediator in cellular balance and highlighting its dual role in promoting healthy aging and driving disease progression.

To conceptualize these processes, the Tai Chi (Yin-Yang) symbol offers a powerful metaphor for the balance between opposing forces that governs cellular behavior. At the heart of cellular growth lies a delicate balance between proliferation and arrest, activity and rest, and life and death. As summarize in Figure 1, in the context of the cell cycle, Yang (the white half of the Tai Chi symbol) represents phases of active growth and division. Cells in the proliferative phase are dynamic, progressing through the G1, S, G2, and M phases as they prepare to replicate and divide, ensuring tissue growth and repair. Stem cells, with their ability to self-renew and differentiate, also inhabit the Yang domain, reflecting their potential for expansion and regeneration. However, this growth must be kept in check by counterbalancing forces, which are represented by Yin (the black half of the symbol). Quiescent cells, residing in the G0 phase, represent the resting state of a cell, where it remains metabolically active but does not proliferate unless stimulated by external signals. Additionally, apoptotic cells, which undergo programmed cell death, embody the concept of controlled degeneration, ensuring that damaged or unnecessary cells are removed to maintain tissue health. In more extreme cases, necrotic cells, which die in an unregulated manner, also fall within the Yin domain, representing a breakdown in cellular control mechanisms.

Within this framework, cancer cells can be placed in the white dot within the black (Yin) half, representing their ability to emerge from a normally controlled, quiescent environment and grow uncontrollably, disrupting the natural balance of cell regulation. These cells break free from the constraints of typical cell cycle arrest, turning what should be a state of rest into a force of unchecked proliferation. In contrast, senescent cells occupy a unique position, symbolized by the black dot within the white (Yang) half. Although they have exited the cell cycle and no longer divide, they remain metabolically active. This makes them a Yin-like force within an otherwise active environment, contributing to tissue remodeling, inflammation, and, in some cases, pathological conditions such as cancer and fibrosis. The pivotal role of senescence in regulating cellular fate underscores its profound impact on both normal aging processes and disease progression.

**Figure 2:**
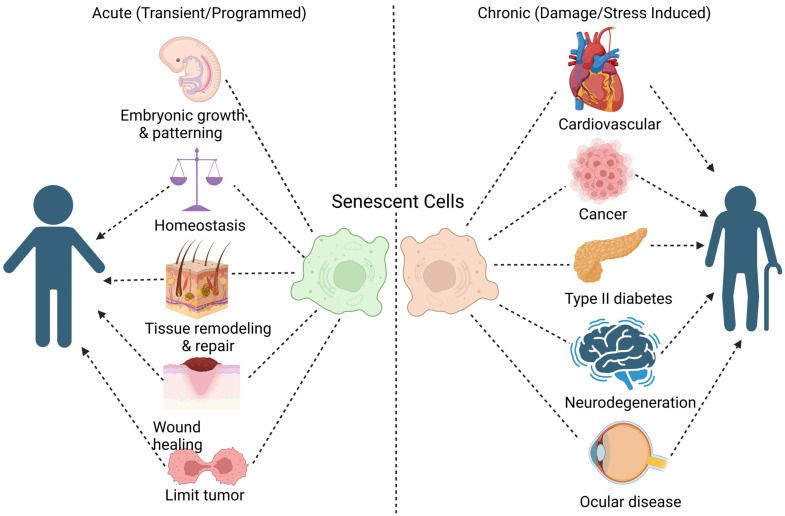
Dual Role of Senescent Cells in Health and Disease. Senescent cells exhibit a dual nature, contributing to both beneficial and detrimental outcomes, depending on the context. On the left, acute (transient or programmed) senescence plays a vital role in promoting homeostasis, embryonic development, tissue remodeling, wound healing, and limiting tumor growth, supporting normal development and repair. On the right, chronic (damage- or stress-induced) senescence is associated with pathological processes, driving age-related diseases such as cardiovascular conditions, cancer, type II diabetes, neurodegeneration, and ocular disease. This figure demonstrates the crucial role of senescence in maintaining tissue health, while highlighting its potential to drive aging and disease when persistently activated.

Throughout our daily activity, our body cells can experience damage from external factors such as sunlight, pollutants, and chemicals, as well as internal factors like metabolism and free radicals [4,5]. Ideally, the cell would undergo a cellular repair mechanism to maintain integrity and metabolic functions. However, when damage is irreparable, cells may undergo apoptosis or programmed cell death to prevent further disturbances to the surroundings [6]. In some cases, however, cells enter a state of permanent cell cycle arrest, becoming unresponsive to growth-promoting signals. This irreversible state, often triggered by DNA damage or cellular stress, is known as cellular senescence. Senescence is a multifaceted biological process with both beneficial and detrimental outcomes, as reflected in Figure 2. In acute, transient contexts, senescence plays critical roles in embryonic development, wound healing, tissue remodeling, and tumor suppression, contributing to homeostasis. These programmed senescent responses are vital for maintaining physiological balance, preventing damaged cells from proliferating uncontrollably, and facilitating tissue repair [7]. However, the chronic accumulation of senescent cells due to persistent stress or damage can lead to pathological consequences [8]. Senescent cells adopt the SASP, secreting pro-inflammatory cytokines, growth factors, and proteases, which can contribute to tissue dysfunction and chronic diseases such as cardiovascular disease [9,10], cancer [11,12,13], type II diabetes [14,15,16,17], neurodegeneration [18,19,20], and age-related ocular diseases [21,22].

Extensive research has shown that various factors contribute to the development of cataracts, including aging, UV exposure, smoking, diabetes, and socioeconomic status [23]. Among these, aging consistently stands out as the primary risk factor for most types of cataracts. Despite this link, research on the relationship between cellular senescence and cataract formation remains limited. The purpose of this review is to explore how senescence affects the lens and drives cataract formation, alongside the emerging therapeutic strategies targeting senescence to promote anti-aging and eye health. By understanding these mechanisms, we can uncover potential interventions to delay or prevent age-related cataracts and improve long-term vision.

## 2. Senescence Overview

### 2.1. History of Senescence

In 1961, a groundbreaking discovery emerged from the meticulous studies conducted by Leonard Hayflick and Paul Moorhead. As they keenly observed cultured human fibroblasts—a type of cell commonly found in connective tissues—they began to discern an intriguing pattern. Over time, these cells seemed to approach an insurmountable boundary, a ceiling to their proliferative abilities. Each cell, irrespective of its initial vigor, appeared to hit a divide-and-grow plateau after a specific number of divisions [24]. This observation contradicted the then-prevailing belief in the endless division potential of cells. The phenomenon they uncovered was subsequently named the “Hayflick limit” in honor of Leonard Hayflick’s pioneering work. An alternative term, the “Hayflick phenomenon”, was also coined to describe this observed cellular behavior. It encapsulated the idea that normal human somatic cells had an intrinsic limit to their division capacity [25]. Once they approached this boundary—capable of roughly 80 divisions—they would no longer divide, transitioning instead into a state known as cellular senescence [26].

### 2.2. Causes of Cellular Senescence

**Figure 3:**
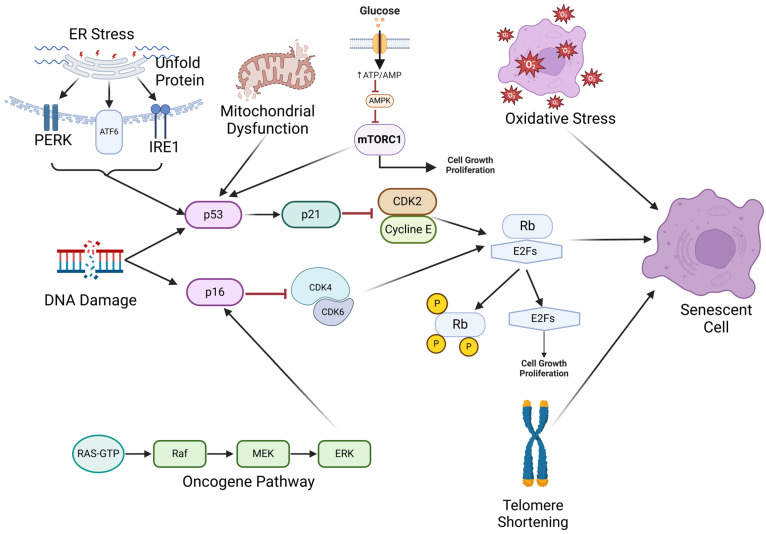
Molecular Pathways Driving Cellular Senescence. The key signaling pathways that lead to cellular senescence in response to various stressors, including ER stress, mitochondrial dysfunction, oxidative stress, DNA damage, oncogene activation, and telomere shortening. DNA damage triggers the p53/p21 and p16/Rb tumor suppressor pathways, resulting in cell cycle arrest through inhibition of CDK4/6 and CDK2, preventing progression at the G1/S checkpoint. Mitochondrial dysfunction and glucose signaling through mTORC1 further reinforce p21 expression, amplifying the senescence response. Oncogene activation, through the RAS pathway (RAS-RAF-MEK-ERK), can trigger p16 expression, further promoting senescence. Telomere shortening promotes Rb phosphorylation, blocking E2F-mediated transcription and halting proliferation. These interconnected pathways ensure that persistent cellular stress culminates in senescence, serving as a protective mechanism against tumorigenesis but contributing to aging and disease when chronically activated. (A black arrow with a pointed end represents activation or promotion, while a red arrow with a blunt end indicates inhibition or suppression).

The major triggers for cellular senescence are summarized in Figure 3. One of the primary causes of cellular senescence is telomere shortening, known as replicative senescence. Telomeres are repetitive nucleotide sequences at the ends of chromosomes that protect them from degradation. With each cell division, telomeres lose around 50–200 base pairs, due to the end-replication problem in DNA synthesis [27]. Once these telomeres attain a critically diminished length, they can elicit a DNA damage response, pushing the cell into a senescent state [28,29]. This mechanism acts as a biological clock, limiting the proliferative capacity of normal somatic cells and preventing genomic instability [30,31,32].

DNA damage and genotoxic stress are significant triggers of cellular senescence, acting as essential protective mechanisms to maintain genomic stability. Such damage can originate from sources including ultraviolet (UV) radiation, ionizing radiation, oxidative stress, and certain chemotherapeutic agents, each capable of inflicting various types of DNA lesions, such as double-strand breaks and base modifications [33]. In response, cells activate DNA damage response (DDR) pathways, with the p53/p21 and p16 tumor suppressor proteins playing central roles in initiating cell cycle arrest [34]. Activation of p53 induces the expression of p21, a cyclin-dependent kinase (CDK) inhibitor, which halts the cell cycle to allow for DNA repair; if the damage is beyond repair, this pathway can push the cell toward senescence or, alternatively, apoptosis [35]. Similarly, the p16 pathway enforces cell cycle arrest by inhibiting cyclin-dependent kinases (CDKs), ensuring that cells with significant DNA damage do not continue to divide [35]. Evidence suggests that p21 acts as an early response factor in the onset of senescence, while p16 is responsible for maintaining the senescent state [36]. This robust response to DNA damage is a vital tumor-suppressive strategy, preventing the propagation of cells with potential oncogenic mutations. However, while senescence effectively curtails the risk of malignant transformation, the accumulation of non-dividing, damaged cells over time can impact tissue function and contribute to age-related pathologies.

Oxidative stress occurs when there is an imbalance between the production of reactive oxygen species (ROS) and the cell’s antioxidant defenses. This imbalance leads to the accumulation of ROS, which can damage cellular components such as lipids, proteins, and DNA [37]. Under normal conditions, the cell activates adaptive responses to mitigate oxidative damage, primarily through the nuclear factor erythroid 2-related factor 2 (Nrf2) pathway [38]. Nrf2 is a transcription factor that, upon activation, translocates to the nucleus and binds to antioxidant response elements (AREs) in the DNA, inducing the expression of various antioxidant enzymes, such as superoxide dismutase (SOD), glutathione peroxidase (GPx), and catalase [39]. These enzymes play essential roles in detoxifying ROS and maintaining cellular redox balance. However, when oxidative stress becomes chronic or excessive due to factors like UV radiation or mitochondrial damage, the antioxidant response can become overwhelmed, resulting in sustained damage and cellular senescence [40,41]. The senescence triggered by oxidative stress involves complex signaling pathways that lead to permanent cell cycle arrest, ensuring that damaged cells do not proliferate [42]. Nrf2 activity often declines with age or under persistent stress conditions, reducing the cell’s capacity to counteract oxidative damage and enhancing the susceptibility to senescence [43,44]. The SASP, which develops as a result of senescence, further exacerbates oxidative stress by promoting pro-inflammatory signaling in the tissue microenvironment [45]. This persistent cycle of oxidative damage and impaired antioxidant response is implicated in aging and age-related diseases.

Mitochondrial dysfunction-associated senescence (MiDAS) occurs when impaired mitochondria disrupt the cellular metabolism and increase the production of ROS [46,47,48]. Mitochondria generate energy through oxidative phosphorylation (OXPHOS), which relies on the electron transport chain (ETC) embedded in the inner mitochondrial membrane. Dysfunctional mitochondria lead to changes in ETC function, causing electron leakage at sites such as Complexes I and III [49,50,51]. This electron leakage results in the partial reduction of oxygen, forming ROS. While ROS at controlled levels serve as signaling molecules, excessive ROS production damages cellular components, including DNA, proteins, and lipids. This oxidative damage activates the DDR pathway, stabilizing tumor suppressor proteins like p53 and inducing cell cycle inhibitors such as p21, which lead to permanent cell cycle arrest and senescence [52,53]. Senescent cells induced by mitochondrial dysfunction exhibit reduced OXPHOS efficiency, altered mitochondrial biogenesis, and often shift to glycolysis (the Warburg effect) [54]. This persistent ROS generation amplifies the SASP, which promotes inflammation and contributes to tissue dysfunction and aging. The continuous vicious cycle of mitochondrial impairment and ROS overproduction reinforces the senescence state, linking mitochondrial health directly to cellular aging and age-related diseases.

Metabolic stress, such as nutrient deprivation or high glucose levels, can induce senescence by altering metabolic signaling pathways [55]. Changes in cellular metabolism are both a cause and consequence of senescence [56]. Senescent cells exhibit altered glucose metabolism, increased glycolysis, and impaired oxidative phosphorylation [57]. Nutrient-sensing pathways involving mechanistic target of rapamycin (mTOR) and AMP-activated protein kinase (AMPK) are implicated in the regulation of senescence [58,59]. Caloric restriction and compounds that mimic its effects have been shown to delay senescence by modulating these metabolic pathways [60,61,62].

Endoplasmic reticulum (ER) stress is another significant factor contributing to cellular senescence. The ER is essential for protein folding and processing; when its capacity is overwhelmed due to an accumulation of misfolded or unfolded proteins, it triggers a cellular response known as the unfolded protein response (UPR). While the UPR initially aims to restore normal function by enhancing the protein folding capacity and degrading misfolded proteins, chronic ER stress can lead to sustained UPR activation. This prolonged stress can induce senescence through pathways involving key regulators like protein kinase R-like endoplasmic reticulum kinase (PERK), activating transcription factor 6 (ATF6), and inositol-requiring enzyme 1 (IRE1) [63]. These pathways may lead to activation of the p53 and p21 pathways, leading to cell cycle arrest and the establishment of a senescent state [64,65,66,67].

Additionally, aberrant oncogene expression can trigger what is termed oncogene-induced senescence (OIS) [1,68,69], serving as a crucial cellular defense mechanism against potential malignant transformation. Oncogenes such as RAS, RAF, and MEK, when aberrantly activated, can lead to hyperproliferative signals that trigger senescence as a safeguard against tumorigenesis [69]. OIS is mediated through the induction of tumor suppressor pathways, particularly those involving p16 and ARF. These pathways inhibit cyclin-dependent kinases and stabilize p53, respectively, thereby halting cell cycle progression and reinforcing the senescence program [70]. While OIS serves as an effective barrier against early tumor development, its dual role in cancer biology is increasingly recognized. As mentioned above, senescent cells can accumulate and secrete a complex mix of pro-inflammatory cytokines, growth factors, and proteases known as the SASP. This secretory profile can remodel the tissue microenvironment, promote angiogenesis, and facilitate epithelial–mesenchymal transition (EMT), inadvertently fostering tumor progression and metastasis [11]. In certain contexts, SASP-driven inflammation can also recruit immune cells, leading to chronic tissue damage and increased genomic instability in neighboring cells, further enhancing the risk of malignant transformation. Thus, while OIS acts as a tumor suppressor in the initial stages of oncogene activation, the persistent presence of senescent cells can paradoxically contribute to tumor progression [71].

### 2.3. Markers for Senescence

**Figure 4:**
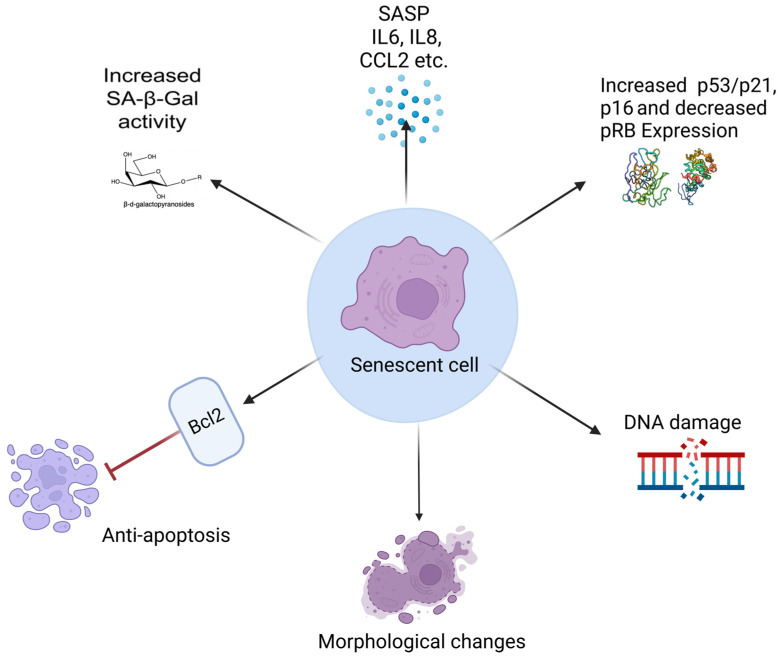
Hallmarks of Cellular Senescence. This figure highlights the key characteristics and molecular features of senescent cells. Senescent cells exhibit increased SA-β-Gal activity, a widely recognized marker of cellular senescence. They display a senescence-associated secretory phenotype (SASP), characterized by the secretion of pro-inflammatory cytokines such as IL-6 and IL-8, chemokines like CCL2, and other factors that influence the surrounding microenvironment. Senescent cells also show upregulation of tumor suppressor pathways, including p53, p21, and p16, along with decreased pRB expression, leading to stable cell cycle arrest. Persistent DNA damage is another feature that contributes to their irreversible growth arrest. Morphological changes, such as cellular enlargement and altered nuclear shape, are common in senescent cells. Despite their arrested state, senescent cells often exhibit resistance to apoptosis through the upregulation of anti-apoptotic factors such as B-cell lymphoma 2 (Bcl-2). (A black arrow with a pointed end represents activation or promotion, while a red arrow with a blunt end indicates inhibition or suppression.)

Identifying senescent cells depends on detecting specific biomarkers; however, no single marker is exclusively definitive. The markers associated with senescence have been thoroughly reviewed by Estela González-Gualda, who emphasized the necessity of multiple hallmarks to confirm a senescent phenotype [72]. Typically, at least three distinct characteristics should be validated: (1) cell cycle arrest, (2) structural changes indicative of senescence, and (3) an additional marker specific to the subtype of senescence being evaluated. Examples of these markers include DNA damage-associated signals, elevated ROS levels, or the upregulation of specific SASP factors. These markers are summarized in Figure 4.

Elevated senescence-associated β-galactosidase (SA-β-gal) activity is the most commonly used marker for cellular senescence [73]. SA-β-gal enzymatic activity is linked to the increased lysosomal content characteristic of senescent cells [74]. The upregulation of the *GLB1* gene, which encodes β-galactosidase, contributes to this elevated activity, reflecting the higher metabolic needs and altered state of senescent cells. Staining with a chromogenic substrate like X-gal produces a blue precipitate, marking SA-β-gal-positive cells. However, SA-β-gal detection should be complemented with other senescence markers, such as p16, p21, or DNA damage indicators, to confirm the senescent phenotype, as SA-β-gal alone is not exclusively definitive as a marker for senescence. It is worth noting that endogenous β-galactosidase in normal cells is enzymatically active at its optimal pH of 4.0. The SA-β-gal assay, however, is performed at a nonoptimal pH of 6.0, to more effectively distinguish between normal and senescent cells. Prolonged incubation times during staining can lead to non-specific positive results, as endogenous β-galactosidase in non-senescent cells may still show residual activity at pH 6.0. To prevent this, it is essential to limit the staining duration to 6–12 h and optimize assay conditions to accurately identify true SA-β-gal activity, thereby avoiding misinterpretation due to background or non-specific staining [75].

The cell cycle arrest is another hallmark of cellular senescence, marked by the presence of protein markers such as p16, p21, and p53, and decreased levels of phosphorylated retinoblastoma protein (pRB) [35]. Two primary regulatory pathways control this process: the p16/RB and p53/p21 pathways. The p16/RB pathway functions by inhibiting CDK4/6, preventing the phosphorylation of RB. When RB remains unphosphorylated, it stays active and binds to E2F, a transcription factor responsible for promoting cell division. This binding stops E2F from activating genes essential for replication, effectively halting the cell cycle. The p53/p21 pathway, on the other hand, is activated in response to stress, such as DNA damage. P53 stimulates the production of p21, which inhibits the CDKs necessary for cell cycle progression. This inhibition keeps RB active, stopping E2F from driving the cell into the next phase of division, thereby arresting the cell cycle.

DNA damage markers play a crucial role in detecting cellular senescence. One of the most widely recognized markers is γ-H2AX, a phosphorylated form of the histone variant H2AX that accumulates at sites of DNA double-strand breaks [76]. The presence of γ-H2AX foci signals an active DNA damage response and is commonly observed in senescent cells where DNA repair is incomplete or ineffective [77]. Another important marker is 53BP1, a DNA damage response protein that colocalizes with γ-H2AX at damage sites and contributes to the recognition and signaling of DNA lesions [78]. The presence of these markers indicates chronic DNA damage, which contributes to the establishment of senescence. Additionally, telomere-associated DNA damage foci (TAF) provide insight into senescence driven by telomere dysfunction, a key feature of replicative senescence. The accumulation of these markers can be visualized through immunofluorescence staining, offering a reliable method for identifying cells that have entered senescence due to sustained DNA damage [79,80]. Together, these markers help distinguish senescent cells from those undergoing transient cell cycle arrest, providing critical insights into cellular aging and the maintenance of tissue homeostasis.

The anti-apoptotic phenotype is another hallmark feature of senescent cells, distinguishing them from other cell states [81]. Senescent cells resist programmed cell death, or apoptosis, through the upregulation of various anti-apoptotic pathways [82]. This resistance is often achieved by activating pathways that inhibit pro-apoptotic factors, including members of the Bcl-2 family, and by overexpressing survival-promoting molecules such as p21 and p16 [83]. This apoptotic characteristic allows these cells to accumulate in tissues, contributing to chronic inflammation and tissue dysfunction.

Morphological changes, including an enlarged, flattened cell shape with increased granularity, are observable under light microscopy and can indicate senescence, though they are not definitive. Additionally, senescent cells often exhibit an increase in the number of vacuoles within their cytoplasm. These vacuoles can vary in size and contribute to the granular appearance of the cytoplasm. The presence of vacuoles is a sign of changes in cellular metabolism and can be associated with the accumulation of senescence-associated β-galactosidase and other by-products. This feature, along with the enlarged and flattened shape, increased granularity, and other markers, helps to distinguish senescent cells from normal, proliferating cells.

Components of the SASP, such as the proinflammatory cytokines interleukin-6 (IL-6) and interleukin-8 (IL-8), are commonly used as markers for cellular senescence. These cytokines indicate the proinflammatory environment established by senescent cells, which can contribute to chronic inflammation and tissue dysfunction. Beyond IL-6 and IL-8, the SASP includes matrix metalloproteinases (MMPs) like MMP-1 and MMP-3, which degrade the extracellular matrix and drive tissue remodeling. Growth factors such as vascular endothelial growth factor (VEGF) and insulin-like growth factor-binding proteins (IGFBPs) also feature prominently in the SASP, influencing angiogenesis and cellular growth. Additional SASP components encompass chemokines like CCL2 (monocyte chemoattractant protein-1) and CXCL1, which recruit immune cells to sites of senescence, altering the local tissue environment. Proteases, including serine proteases and cathepsins, modulate extracellular structures and signaling pathways, impacting neighboring healthy cells. ROS and signaling molecules like amphiregulin (AREG) are also part of the SASP, activating various cellular pathways. Collectively, these diverse factors play a critical role in modifying the tissue microenvironment and influencing surrounding cell behavior, providing a comprehensive profile that helps identify and characterize the senescent state beyond morphological changes.

## 3. Human Lens and Lens Epithelial Cells (LECs)

The human eye contains a lens that focuses light onto the retina, producing clear images of objects at varying distances. At birth, the lens weighs around 65 mg and grows to approximately 160 mg by age 10. After that, growth slows, and by the age of 90, the average lens weight is around 250 mg [84,85,86,87]. The lens maintains transparency through a combination of anatomical and physiological adaptations. Anatomically, it is avascular, preventing light obstruction by blood vessels. The lens comprises the lens capsule, epithelium, and fibers. The lens capsule, a transparent and elastic outer layer, serves as a selective barrier that facilitates nutrient and waste exchange, ensuring a stable internal environment. The lens epithelium, composed of a single layer of metabolically active LECs, produces essential biomolecules and ATP. As these LECs migrate toward the lens equator, they differentiate into lens fiber cells (LFCs), undergoing significant elongation and losing their organelles, including nuclei and mitochondria, to create an organelle-free zone (OFZ) that minimizes light scattering [84,87]. The high concentration of crystallin proteins in both LECs and LFCs, along with the actin cytoskeleton’s role in LFC elongation and differentiation, further contributes to the lens’s optical clarity. The formation of OFZ and precise protein arrangement ensure that light passes through the lens without absorption or scattering, maintaining clear vision [87].

**Figure 5:**
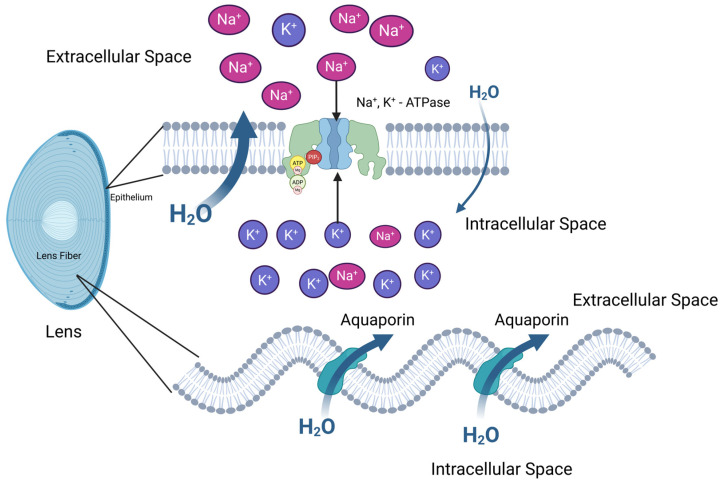
Function of Lens Epithelial Cells in Ion and Water Homeostasis. This figure highlights the critical role of lens epithelial cells (LECs) in regulating ion and water balance to maintain lens transparency and function. LECs actively maintain Na^+^ and K^+^ gradients through Na^+^/K^+^-ATPase, which pumps Na^+^ out of the cell and K^+^ in, preventing osmotic imbalance and cellular swelling. Aquaporins facilitate the movement of water (H₂O) across cell membranes, ensuring proper hydration and fluid exchange between intracellular and extracellular spaces. This coordinated regulation of ion transport and water flow by LECs preserves lens homeostasis, preventing cataract formation and supporting lens clarity.

Apart from anatomical factors, physiological factors also impact lens transparency. As shown in Figure 5, the Na^+^/K^+^-ATPase pump in LECs and LFCs keeps the electrolyte and water balance, maintaining a low sodium concentration inside the lens. This creates a hypoosmotic intracellular environment, causing fluid to flow out of the lens, which is essential for lens transparency. Additionally, aquaporin 0, a water channel highly expressed in the lens fiber cell membrane, helps maintain the lens’s dehydrated state [88].

Furthermore, the lens’s antioxidative defense system includes small molecules like reduced glutathione (GSH), which neutralizes ROS and is regenerated through enzymatic processes to maintain its antioxidant capacity. In addition to GSH, antioxidant enzymes such as catalase, SOD, glutaredoxin (Grx), and thioredoxin (Trx) play essential roles in detoxifying harmful oxidative byproducts like hydrogen peroxide and superoxide radicals. This defense system protects lens proteins from oxidative stress, which is especially important given the lens’s exposure to environmental factors like UV light, thereby preserving transparency and preventing protein modification. [39,89,90,91].

Despite the lens’s intricate physiological mechanisms for maintaining transparency, these defenses gradually weaken with age. Over time, the cumulative effects of oxidative stress and protein damage can lead to the clouding of the lens, resulting in cataract formation. Cataracts occur when normally clear lens fibers and proteins begin to aggregate and scatter light, creating a frosted or blurry effect, much like looking through a foggy window. While many cataracts develop slowly and may not initially impair vision, they can progressively worse, eventually causing significant visual impairment and even reduce life expectancy if left untreated [92,93]. According to CDC statistics, cataracts are the leading cause of vision loss worldwide [94]. According to the NIH 2014 Eye Disease Fact Sheet, an estimated 24 million Americans aged 40 and older (17.2%) have cataracts in one or both eyes, and 6.1 million (5.1%) have undergone surgery to remove them. Additionally, the Eye Care Surgeons and Associates reported that by age 65, more than 90% of Americans and 50% of people globally will develop cataracts [94,95].

Cataracts can be classified into several types, with the most common being nuclear sclerotic, cortical, and posterior subcapsular [96,97,98]. Nuclear sclerotic cataract affects the central part of the lens (the nucleus) and typically develops with age. It causes distant vision to become blurry, and as it progresses, the lens may turn yellow or even brown, further impairing vision. Cortical cataract forms along the outer edge of the lens, creating wedge-shaped opacities that spread inward. This type is often associated with diabetes and can cause glare and night vision issues. Posterior subcapsular cataract develops at the back of the lens and is often linked to steroid use, diabetes, or radiation exposure. It affects near vision and can cause glare, particularly in bright light or at night. Unlike other types, posterior subcapsular cataracts tend to progress more rapidly, making early detection important.

## 4. LECs and Senescence

### 4.1. LEC Senescence and Cataract

Several studies have utilized human tissues from clinical setting and found possible relationships between increased LEC senescence and different types of cataract. For example, Yao and colleagues examined lens capsules from cataract patients of various age groups and observed that markers associated with progenitor cell function, such as Sox2, Abcg2, and Ki67, significantly decreased with age and were nearly absent in individuals over 60. This depletion of progenitor cells was paralleled by an increase in senescence-associated β-galactosidase (SA-β-gal) staining, a well-established senescence marker, particularly in cortical cataracts. The study provided strong evidence linking the decline in regenerative capacity of LECs due to senescence to the severity of cortical cataracts, a type closely associated with the aging process [99].

Another study revealed how alterations in the extracellular environment of LECs contribute to senescence by disrupting normal cellular signaling and structural integrity. In age-related cataract patients, aged LECs show increased cellular stress and senescence as a result of matrix imbalances and changes in intercellular signaling, specifically within pathways that exacerbate stress responses in senescent cells. When oxidative stress is induced in cultured LECs, they respond by adopting senescent characteristics, such as an enlarged, flattened morphology and higher SA-β-gal activity, mirroring changes observed in cataractous lenses. The study demonstrated that age-related modifications in the lens matrix drive the cells further into a senescent state, limiting their functional capacity [100]. Thus, approaches aimed at restoring a balanced matrix environment and reducing stress signaling in LECs could help delay the onset of senescence, highlighting the matrix as an essential factor in maintaining cellular health within the lens.

In a third study, Zhou and colleagues examined human LECs from lenses with varying degrees of cataract severity, to explore the relationship between cataract progression and cellular senescence. They found that more advanced cataracts showed higher levels of senescent markers, particularly SA-β-gal staining, which is indicative of cellular aging. In cataracts of greater severity, senescent cells accumulated more extensively, suggesting a strong correlation between the degree of lens opacity and the presence of senescent LECs. This gradient of senescence in correlation with cataract severity highlights that as LECs undergo senescence, they not only lose regenerative potential but also increasingly contribute to the opacity characteristic of cataractous lenses. The study provided critical evidence that addressing senescence in LECs could potentially prevent or slow the progression of cataract, especially in early-stage cataracts where interventions may have the most substantial impact [101].

### 4.2. Causes of LEC Senescence

As mentioned above, LECs serve several essential functions that contribute to maintaining lens health and ensuring proper vision. LECs are metabolically active, producing ATP and essential biomolecules that support the overall transparency and function of the lens. As progenitor cells for LFCs, LECs migrate toward the lens equator and differentiate into LFCs, undergoing organelle elimination to create an organelle-free zone that minimizes light scattering and maintains lens clarity. They play a critical role in lens regeneration and repair, aiding in the recovery from minor damage and promoting long-term lens health. Additionally, LECs synthesize crystallin proteins, which are crucial for the lens’s refractive properties and optical clarity. They also regulate the movement of nutrients and waste between the lens and surrounding fluids, ensuring a stable internal environment. Importantly, LECs contribute to the lens’s antioxidant defense by neutralizing oxidative stress and preventing cellular damage, thereby preserving lens transparency and function [102,103].

**Figure 6:**
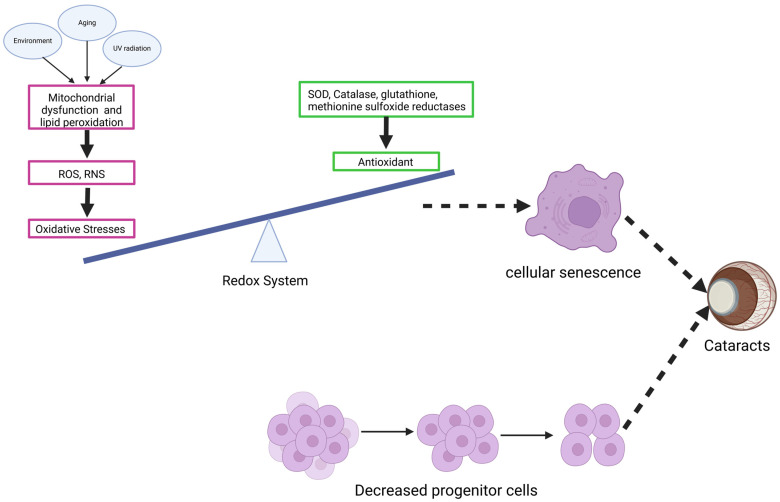
Cellular Senescence and Cataract Formation in the Lens. Oxidative stress drives cellular senescence in lens epithelial cells (LECs), contributing to cataract formation. Environmental factors such as aging and UV radiation trigger mitochondrial dysfunction and lipid peroxidation, leading to the production of reactive oxygen species (ROS) and reactive nitrogen species (RNS). This results in oxidative stress. The balance between oxidative stress and the redox system, maintained by antioxidants like SOD, catalase, glutathione, and methionine sulfoxide reductases, determines the cellular fate. Excessive oxidative stress disrupts this balance, inducing cellular senescence in LECs, reducing progenitor cell populations, and promoting cataract development.

While LECs are essential for maintaining lens health and function, they are not immune to age-related changes. One significant challenge to the longevity and functionality of LECs is cellular senescence. As shown in Figure 6, LEC senescence is primarily driven by oxidative damage due to continuous exposure to external stressors like UV radiation and internal factors such as mitochondrial ROS production. Over time, these oxidative stressors disrupt normal redox homeostasis, leading to the progressive accumulation of ROS and reactive nitrogen species (RNS) and triggering a cascade of molecular changes that induce senescence.

Another mechanism underlying LEC senescence is oxidative modification of key cellular components, including DNA, proteins, and lipids. Oxidative stress damages nuclear and mitochondrial DNA, impairing repair mechanisms and causing persistent DNA damage. This activates the DDR pathway, which upregulates markers such as γ-H2AX and p53, leading to cell cycle arrest [104]. The p16/Rb and p21/p53 pathways, activated by oxidative stress, further enforce this arrest by inhibiting CDKs. These pathways collectively contribute to a senescent phenotype marked by permanent growth arrest and metabolic reprogramming.

Mitochondrial dysfunction also plays a significant role in exacerbating LEC senescence. Damaged mitochondria produce excess ROS and reduce ATP synthesis, creating a feedback loop of oxidative damage that accelerates cellular aging [105]. This decline in mitochondrial function contributes to the energy deficits and metabolic changes commonly observed in senescent cells [106].

Additionally, the loss of antioxidant defenses in aging LECs—marked by reduced activity of SOD, catalase, Grx, and GSH—leaves the cells vulnerable to oxidative damage [90,91]. Impaired repair of oxidatively modified proteins due to decreased levels of methionine sulfoxide reductases leads to protein misfolding and aggregation, further driving cellular dysfunction [107]. Furthermore, a recent study demonstrated that primary LECs isolated from one-month-old *Grx1/Grx2* double knockout mice exhibited premature senescence. In contrast, LECs from wild-type (WT) mice of the same age did not display any signs of senescence, highlighting the critical role of Grx1 and Grx2 in maintaining cellular health at an early stage [108]. Grx1 and Grx2 are Grxs located in the cytosol and mitochondria, respectively, and are essential for maintaining redox homeostasis by facilitating the reduction of protein disulfides and mixed disulfides with GSH. The absence of both *Grx1* and *Grx2* disrupts the cellular capacity to repair oxidatively damaged proteins, leading to the accumulation of oxidized cysteine residues within proteins. This accumulation promotes cellular dysfunction and triggers premature senescence in LECs. These findings highlight the critical role of Grx-mediated protein repair mechanisms in protecting against oxidative stress-induced cellular aging.

The impact of senescence extends beyond individual LECs. Senescent cells accumulate and lose their proliferative capacity, undergoing significant morphological and functional changes that compromise lens transparency. Prolonged oxidative stress also diminishes the regenerative potential of the lens by affecting progenitor cells, which are crucial for replenishing aging or damaged LECs [99]. These progenitor cells become prone to redox imbalances and enter a senescent state, limiting their differentiation and self-renewal capacity. This reduction in progenitor cell function exacerbates the accumulation of senescent LECs, forming a cycle that leads to impaired lens repair and accelerated lens opacification [109]. As senescent cells accumulate, they disrupt lens homeostasis and contribute to cataractogenesis. The decline in both LEC and progenitor cell function results in reduced capacity for lens maintenance and regeneration, fostering a pro-inflammatory environment that further promotes oxidative damage. The interplay of oxidative stress, cellular senescence, mitochondrial dysfunction, and weakened antioxidant defenses directly influences the pathogenesis of cataracts.

In summary, LEC senescence is driven by a complex network of oxidative stress, DNA damage, mitochondrial dysfunction, and reduced antioxidant defenses. These cellular and molecular changes diminish the lens’s regenerative capacity and accelerate cataract formation. Understanding these pathways is essential for developing therapeutic strategies to delay LEC senescence and prevent age-related cataracts.

### 4.3. To Apoptosis or Not to Apoptosis?

In the study of age-related cataract formation, understanding the mechanisms of LEC death is critical. For years, apoptosis was hypothesized as a major contributor to cataract formation. Early research, such as the studies by Li et al., proposed that LEC apoptosis was a primary mechanism in both animal and human cataracts, showing increased terminal deoxynucleotidyl transferase dUTP nick end labeling (TUNEL)-positive cells and DNA fragmentation in cataractous lenses [110]. This led to a theory that blocking apoptosis might slow or prevent cataract formation.

However, later findings have challenged this apoptosis-centered view. Dr. Beebe’s group critically evaluated cell death in LECs from human cataract specimens, using a combination of TUNEL assays, cell proliferation markers, and cell density measurements. They found minimal evidence of apoptosis in cataractous lenses; instead, TUNEL staining suggested necrosis, likely from surgical handling or oxidative insult, rather than apoptosis. Furthermore, they observed stable cell density in cataractous lenses, with no compensatory cell proliferation, which would be expected if significant cell death were occurring. These results suggest that apoptosis may not play a prominent role in human cataract formation, contradicting earlier studies and calling for a reevaluation of LEC death mechanisms in cataract [111].

Building on Dr. Beebe’s findings, we propose an additional explanation grounded in the biology of cellular aging: rather than undergoing cell death, LECs in cataractous lenses may, at least in part, be entering a state of senescence. A hallmark of senescent cells is their resistance to apoptosis. These cells often exhibit elevated Bcl-2 levels and lack caspase-3 activation, protecting them from programmed cell death. Senescence, therefore, could explain the stable LEC density observed in cataractous lenses and the lack of apoptotic markers, as these cells resist apoptotic signals and persist in the tissue. In age-related cataracts, LECs progressively accumulate damage and become dysfunctional through senescence, rather than apoptosis, contributing to lens opacity, while maintaining overall cell density.

This shift in perspective from apoptosis to senescence opens new avenues for therapeutic approaches. Targeting senescence pathways, either through senolytic drugs that clear senescent cells or compounds that modulate the SASP, could potentially delay cataract formation by addressing the underlying cellular changes rather than simply preventing cell death. Future studies exploring senescence markers in LECs and the role of the SASP in cataractogenesis will be essential for confirming this hypothesis and refining our approach to managing age-related cataract.

## 5. Senotherapeutics

**Figure 7:**
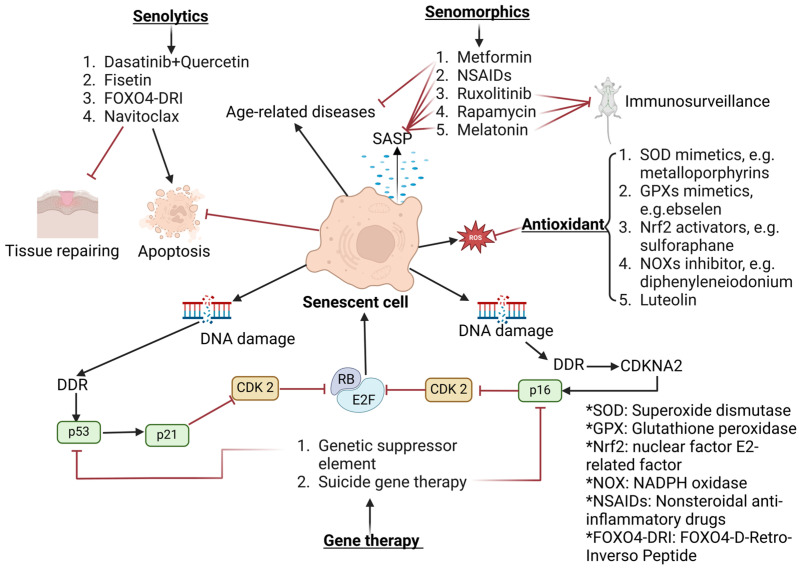
Senotherapeutics for Targeting Senescent Cells. Senotherapeutics aim to manage cellular senescence by either eliminating senescent cells or modifying their behavior to mitigate age-related diseases. Senolytics, such as Dasatinib + Quercetin, Fisetin, FOXO4-DRI, and Navitoclax, selectively induce apoptosis in senescent cells, aiding tissue repair and reducing inflammation. Senomorphics, such as Metformin, NSAIDs, Ruxolitinib, Rapamycin, and Melatonin, suppress SASP, limiting the pro-inflammatory effects of senescent cells, without eliminating them. Antioxidants like SOD mimetics, GPX mimetics, and NRF2 activators reduce oxidative stress, protecting cells from senescence. Immunosurveillance further supports the clearance of senescent cells by enhancing immune responses. Gene therapy approaches targeting CDK2, p53, p21, and p16 pathways offer additional strategies to control senescence, promoting genomic stability and cellular homeostasis. These interventions collectively aim to restore tissue function and delay the onset of age-related diseases. (A black arrow with a pointed end represents activation or promotion, while a red arrow with a blunt end indicates inhibition or suppression).

The major senotherapeutics for targeting senescent cells are summarized in Figure 7. As previously discussed, cellular senescence significantly contributes to aging and the pathogenesis of numerous age-related diseases. The accumulation of senescent cells is frequently observed at pathogenic sites in several major chronic conditions, including cataracts, Alzheimer’s disease, cardiovascular disease, osteoporosis, diabetes, chronic kidney disease, and liver cirrhosis [112]. The persistence of these cells is particularly problematic, as they release a range of pro-inflammatory factors known collectively as the SASP [113]. The SASP can damage surrounding tissues and foster a pro-inflammatory environment, effectively making aging a transmissible condition. In this context, one senescent cell can influence neighboring cells, promoting their transition to a senescent state as well [24]. Given this, therapeutic interventions aimed at reducing the senescent cell burden or mitigating their adverse effects have become a key focus to extend a healthy lifespan and delay the onset of age-related diseases. Therapeutic strategy has led to the emergence of senotherapeutics, a class of therapies designed to target senescent cells and mitigate their harmful effects. Senotherapeutics can be categorized into two primary types: senolytics, which pharmacologically induce the elimination of senescent cells, and senomorphics, which modulate the SASP and other markers of cellular senescence to mitigate their negative effects, without removing the cells themselves [114].

Senescent cells can develop resistance to apoptosis, often through the upregulation of anti-apoptotic pathways that shield them from self-induced cytotoxic effects. This resistance enables senescent cells to persist within tissues, where they may contribute to degenerative diseases by impairing tissue function and inducing local inflammation. To counter this, senolytic drugs have been developed to selectively induce apoptosis in senescent cells, while sparing non-senescent cells, thereby preserving overall tissue health [115]. Pioneering studies by Kirkland and colleagues proposed that targeting multiple anti-apoptotic pathways simultaneously, rather than a single pathway, may improve the effectiveness of senolytic therapies. For instance, they found that a combination of dasatinib, an anticancer drug, and quercetin, a natural flavonoid, successfully induced apoptosis in senescent cells, without harming non-senescent cells [116]. Another promising compound, fisetin—also a flavonoid—has demonstrated senolytic effects by acting on specific anti-apoptotic networks, suggesting its potential as a therapeutic agent in managing age-related diseases [117,118,119,120]. Other than small molecule compounds, Forkhead Box O4-D-Retro-Inverso peptide (FOXO4-DRI) is a synthetic peptide developed as a senolytic agent that selectively induces apoptosis in senescent cells. It functions by disrupting the interaction between the transcription factor FOXO4 and the tumor suppressor protein p53 within these aged cells. In normal senescent cells, FOXO4 binds to p53, sequestering it in the nucleus and preventing it from initiating apoptosis. FOXO4-DRI competes with endogenous FOXO4 to bind to p53, effectively releasing p53 to trigger the apoptotic pathway. This targeted mechanism allows FOXO4-DRI to eliminate senescent cells, without harming healthy cells, thereby reducing the accumulation of dysfunctional cells that contribute to aging and age-related diseases. Preclinical studies have shown that treatment with FOXO4-DRI can alleviate symptoms of aging and improve organ function in animal models, highlighting its potential as a therapeutic agent in combating age-associated conditions [121,122,123]. However, Kowald et al. cautioned that while senolytics may reduce the burden of senescent cells, repeated treatments could inadvertently accelerate the formation of new senescent cells, potentially compromising the body’s regenerative capacity [124]. This possibility underscores the need for a balanced and carefully monitored approach in the clinical use of senolytic therapies.

In contrast to senolytics, senomorphics offer an alternative approach by targeting the SASP to mitigate the detrimental effects of senescent cells, without eliminating them. Metformin, a well-established antidiabetic medication, has gained considerable attention as a senomorphic agent, with potential benefits extending beyond glycemic control [125]. As a senomorphic drug, metformin modulates the detrimental effects of cellular senescence, without necessarily eliminating senescent cells [126]. It achieves this by influencing key metabolic and signaling pathways associated with aging. Studies have shown that metformin activates AMPK, which enhances the cellular energy balance and inhibits the mechanistic target of mTOR pathway—both critical regulators of aging and longevity [127,128,129]. By reducing oxidative stress and inflammation, metformin mitigates the SASP, thereby decreasing chronic low-grade inflammation linked to age-related diseases. Clinical and epidemiological evidence suggests that metformin use is associated with a reduced incidence of various age-related conditions, including type II diabetes, cardiovascular diseases, neurodegenerative disorders, and certain types of cancer [130,131]. Other senomorphic agents, such as ruxolitinib [132] and rapamycin [133], have demonstrated efficacy in suppressing inflammatory SASP factors, potentially providing therapeutic benefits for conditions worsened by chronic inflammation [134]. Additionally, melatonin—a hormone with anti-aging properties—exerts senomorphic effects by downregulating SASP gene expression, adding to the growing arsenal of senomorphic therapies [135,136,137]. Despite their promise, senomorphic therapies pose unique challenges, due to the dual roles of SASP factors, which are involved in immune surveillance and tumor suppression. Modulating the SASP could unintentionally impact these physiological functions, raising concerns about the long-term safety and efficacy of senomorphic treatments, especially in clinical settings, where immune balance is essential.

Antioxidants also play a critical role in senotherapeutics by combating the oxidative stress that often accompanies cellular senescence. Compounds like SOD mimetics [138], GPx mimetics [139], and Nrf2 activators (e.g., sulforaphane) can reduce oxidative damage within cells, thus preventing the accumulation of DNA damage that promotes senescence [140]. Furthermore, NOX inhibitors, such as diphenyleneiodonium, target NADPH oxidase, a key enzyme in producing the ROS associated with SASP [141]. By lowering oxidative stress, these antioxidants help minimize DNA damage, suppress SASP production, and alleviate inflammation, thereby promoting healthier cellular function [142].

Gene therapy is emerging as a promising senotherapeutic strategy to address the genetic and molecular pathways underlying cellular senescence. This approach leverages tools such as genetic suppressor elements and suicide gene therapy to target and repair the mechanisms driving senescence [143]. By modulating genes involved in cell cycle regulation—such as CDK2 and the tumor suppressor RB (retinoblastoma protein)—gene therapy has the potential to delay the onset of senescence or facilitate the repair of damaged DNA, thereby restoring cellular function [144]. In more specialized applications, suicide gene therapy enables the selective elimination of senescent cells by activating intrinsic apoptosis pathways, providing a precise method to reduce the senescent cell burden, without harming healthy cells. This dual capability—repairing cellular damage and inducing apoptosis in irreversibly senescent cells—positions gene therapy as a highly targeted and adaptable tool in the senotherapeutic field. By addressing the root genetic drivers of cellular aging, gene therapy offers a sophisticated and potentially transformative approach to mitigating age-related diseases, including cataracts and other degenerative conditions linked to cellular senescence.

## 6. Senotherapeutics for the Lens

As summarized in Table 1, we have outlined the key senotherapeutics for cataract prevention. Senolytics, which selectively induce apoptosis in senescent cells while sparing healthy ones, have emerged as a promising strategy for reducing the oxidative stress and inflammation associated with cataract formation. By clearing senescent LECs, these drugs can potentially address the cellular damage that contributes to cataract onset. Among the key senolytics being investigated are dasatinib and quercetin, a combination that has demonstrated broad efficacy across various tissues. This approach may hold particular relevance in reducing the burden of senescent cells in the lens, thereby delaying cataract progression. In addition to dasatinib and quercetin, other senolytics such as fisetin and navitoclax have garnered attention for their potential in cataract prevention. Fisetin, a natural flavonoid, not only targets senescent cells but also reduces oxidative stress markers, making it a dual-action candidate for preserving lens clarity. Similarly, navitoclax, known for its role in disrupting anti-apoptotic pathways, offers another avenue for clearing damaged LECs and mitigating cataract risk.

While senolytics directly eliminate senescent cells, senomorphics provide a complementary, non-destructive strategy by modulating the harmful effects of the SASP. This pathway is particularly advantageous in tissues like the lens, where maintaining cell populations is essential. By suppressing SASP-induced inflammation and oxidative damage, senomorphics can stabilize lens function and potentially slow cataract progression. Among the senomorphic agents under investigation, metformin stands out for its established anti-aging properties. By reducing SASP expression and enhancing cellular resilience, metformin may play a critical role in preserving lens transparency. Similarly, rapamycin, which inhibits the mTOR pathway, helps suppress SASP factors and alleviate cellular stress, contributing to cataract prevention. Melatonin, known for its anti-inflammatory and antioxidative effects, has also demonstrated senomorphic potential, further broadening the arsenal of cataract-preventive interventions.

Emerging preclinical studies have underscored the potential of senotherapies in cataract prevention. Using a rat model of cataracts induced by D-galactose, researchers tested a combination of dasatinib and quercetin, known for its senolytic properties, as well as rapamycin, an inhibitor of inflammatory signals from senescent cells [104]. Both treatments initially reduced cataract markers and LEC senescence indicators, suggesting their potential to delay early cataract development. However, their effects diminished in later stages, indicating the need for further investigation into dosing and treatment timing. This work highlighted senotherapy’s promise as a potential strategy for early cataract intervention.

Building on its senolytic properties, fisetin, a natural flavonoid, exhibits anti-cataract properties by targeting mechanisms related to oxidative stress, inflammation, and cellular senescence. In diabetic conditions, oxidative stress and the accumulation of advanced glycation end products accelerate cataract formation. Fisetin mitigates oxidative damage by scavenging free radicals and enhancing cellular antioxidant defenses. Additionally, it reduces inflammation by inhibiting key pro-inflammatory pathways, including nuclear factor kappa-light-chain-enhancer of activated B cells (NF-κB), which otherwise contribute to lens damage in diabetes. Furthermore, fisetin’s senolytic properties help clear the senescent cells that accumulate in lens tissues, thus preventing the progression of cataractogenic changes. Through these combined actions, fisetin supports lens transparency and delays cataract progression in stress-induced and aging-related models [145].

Luteolin, like fisetin, is a flavonoid known for its eye health benefits that demonstrates anti-cataract effects primarily through its antioxidant and anti-apoptotic properties. In a selenite-induced cataract model, luteolin administration enhanced the activity of antioxidant enzymes, reducing the ROS that cause oxidative damage to the lens [146]. This antioxidant boost helps maintain lens clarity by counteracting oxidative stress, a key factor in cataractogenesis. Additionally, luteolin stabilizes lens membrane integrity and maintains ionic balance by increasing Ca^2+^-ATPase activity, which regulates the calcium levels crucial for cellular stability. Its inhibition of caspase-3 activity and expression indicates a reduction in apoptosis, preserving lens cell viability. These combined actions suggest that luteolin supports lens health by protecting against oxidative stress, preventing calcium imbalance, and reducing cell death pathways.

As cells age, LECs show increased markers of senescence, such as p21 and p53, which contribute to the degradation of lens clarity in age-related cataract. Metformin counters this by activating the AMPK pathway, which is typically diminished in aged cells. AMPK activation reduces cellular senescence through enhanced autophagy, a vital process that clears damaged cellular components and supports cellular health. By restoring autophagic flux and suppressing senescence markers, metformin addresses the cellular aging processes in LECs, positioning it as a potential therapy for delaying cataract progression by targeting the root causes of cellular aging [147,148].

Similarly, melatonin’s protective role against cataract is focused on its ability to counteract cellular senescence by inhibiting ferroptosis—a cell death pathway often linked to senescence in oxidative stress conditions. UVB-induced oxidative stress in lens epithelial cells typically accelerates senescence through iron accumulation and lipid peroxidation, leading to DNA and protein damage. Melatonin counters this process by activating SIRT6, a longevity-associated enzyme that regulates senescence. Through the SIRT6/p-Nrf2/GPX4 pathway, melatonin enhances antioxidant defenses and activates antiferroptosis pathways, leading to a reduction in senescence markers. Additionally, SIRT6 activation stabilizes cellular iron balance via the nuclear receptor coactivator 4/ferritin heavy chain 1 (NCOA4/FTH1) pathway, preventing the iron-induced oxidative stress that accelerates senescence. By modulating these pathways, melatonin effectively limits cellular aging in the lens, highlighting its potential as a senotherapeutic for delaying cataract progression through both antioxidant and iron-regulatory mechanisms [149].

## 7. Conclusions and Future Directions

Cellular senescence plays a dual role in health and disease, acting as both a guardian against uncontrolled proliferation and a driver of age-related pathologies, including cataract formation. The intricate interplay between oxidative stress, mitochondrial dysfunction, and chronic inflammation underscores the complexity of senescence in LECs. As the primary cellular component maintaining lens transparency, the progressive senescence of LECs represents a critical factor in age-related cataractogenesis. Advances in senotherapeutics may offer promising strategies to mitigate LEC senescence, either by eliminating senescent cells through senolytics or modulating the harmful effects of the SASP with senomorphics. Natural compounds like fisetin, luteolin, and metformin, along with innovative therapies such as FOXO4-DRI and gene editing, highlight the growing potential for targeted interventions to delay cataract progression.

Future research on cellular senescence in cataract formation holds significant potential to uncover novel therapeutic strategies aimed at delaying or preventing lens opacity. A deeper understanding of the molecular drivers of LEC senescence, particularly the role of oxidative stress, mitochondrial dysfunction, and protein aggregation, will be essential for developing targeted interventions. Investigating the interplay between SASP factors and changes in the lens microenvironment could provide insights into how chronic inflammation accelerates cataract progression. Expanding high-throughput screening of natural and synthetic compounds to identify agents that mitigate senescence, enhance antioxidant defenses, or inhibit protein misfolding is another promising avenue. Additionally, exploring the role of genetic and epigenetic regulators in lens senescence may reveal novel biomarkers for early detection and intervention. Future studies should also focus on the systemic impact of aging and how it influences lens health, potentially uncovering links between systemic senescence and ocular diseases. By integrating these approaches, senescence research could pave the way for innovative treatments that preserve lens transparency and prevent age-related cataracts.

## Figures and Tables

**Table 1:** Summary of senotherapeutics for the lens.

Agent	Senotherapeutics	Mechanism of Action	Therapeutics	Refs.
Dasatinib + Quercetin	Senolytics	Induces apoptosis in senescent cells	Reduces senescent cell burden in the lens, delaying cataract progression	[104]
Fisetin	Senolytics	Targets senescent cells and reduces oxidative stress marker	Enhances lens clarity by decreasing oxidative stress and inflammation	[145]
Luteolin	Senolytics	Antioxidant, antiapoptotic, and calcium homeostasis regulator	Maintains lens clarity, stabilizes membrane integrity, and prevents LEC apoptosis	[146]
Metformin	Senomorphics	Activates AMPK, suppresses SASP and enhances autophagy	Reduces SASP factors, enhances cellular resilience, mitigates oxidative stress, and preserves lens transparency	[147,148]
Melatonin	Senomorphics	Antioxidant and anti-inflammatory	Inhibits oxidative damage and ferroptosis, supporting lens health and transparency	[149]

## Data Availability

All data are available from the corresponding author by request.

## Abbreviations

The following abbreviations are used in this manuscript:

AMPK	AMP-activated protein kinase
ARE	Antioxidant response element
ATF6	Activating transcription factor 6
Bcl-2	B-cell lymphoma 2
CDK	Cyclin-dependent kinase
CDKs	Cyclin-dependent kinases
DDR	DNA damage response
DRI	D-retro-inverso
ETC	Electron transport chain
FTH1	Ferritin heavy chain 1
FOXO4	Forkhead box O4
GSH	Glutathione
GPx	Glutathione peroxidase
Grx	Glutaredoxin
IL-6	Interleukin-6
IL-8	Interleukin-8
LEC	Lens epithelial cell
LECs	Lens epithelial cells
MMPs	Matrix metalloproteinases
mTOR	Mechanistic target of rapamycin
MiDAS	Mitochondrial dysfunction-associated senescence
NF-κB	Nuclear factor kappa-light-chain-enhancer of activated B cells
Nrf2	Nuclear factor erythroid 2-related factor 2
OFZ	Organelle-free zone
OIS	Oncogene-induced senescence
OXPHOS	Oxidative phosphorylation
p16	Cyclin-dependent kinase inhibitor 2A (CDKN2A)
p21	Cyclin-dependent kinase inhibitor 1A (CDKN1A)
p53	Tumor protein p53
PERK	Protein kinase R-like endoplasmic reticulum kinase
RB	Retinoblastoma protein
RNS	Reactive nitrogen species
ROS	Reactive oxygen species
SASP	Senescence-associated secretory phenotype
SA-β-gal	Senescence-associated beta-galactosidase
SOD	Superoxide dismutase
TAF	Telomere-associated DNA damage foci
TUNEL	Terminal deoxynucleotidyl transferase dUTP nick end labeling
UPR	Unfolded protein response
UV	Ultraviolet
VEGF	Vascular endothelial growth factor
WT	Wild-type
